# Disparities in the uptake of HPV-related cancer prevention strategies among women by HIV status in Puerto Rico

**DOI:** 10.1016/j.gore.2025.101687

**Published:** 2025-02-03

**Authors:** Marievelisse Soto-Salgado, Melisa Ramos-Sepúlveda, Jeslie M. Ramos-Cartagena, Maribel Tirado-Gómez, Humberto M. Guiot, Cristina Muñoz-Masso, Lorena González-Sepúlveda, Ana Patricia Ortiz

**Affiliations:** a University of Puerto Rico (UPR) Comprehensive Cancer Center, Division of Cancer Control and Population Sciences, San Juan, PR, United States; b UPR Comprehensive Cancer Center, Cancer Prevention and Control Research (CAPAC) Training Program, San Juan, PR, United States; c UPR Comprehensive Cancer Center, Division of Clinical and Translational Cancer Research, San Juan, PR, United States; d UPR Medical Sciences Campus, School of Medicine, Department of Medicine, San Juan, PR, United States; e UPR Medical Sciences Campus, School of Medicine, Department of Microbiology and Medical Zoology, San Juan, PR, United States; f UPR Medical Sciences Campus, Graduate School of Public Health, Department of Biostatistics and Epidemiology, San Juan, PR, United States

**Keywords:** HIV, Cervical Pap screening, Anal Pap screening, HPV vaccine, Hispanics, Puerto Rico

## Abstract

**Background:**

Women living with HIV (WLWH) are at increased risk of persistent HPV infection and HPV-related cancers than persons without HIV. We aimed to describe the utilization of primary and secondary prevention strategies for HPV-related cancers among women by HIV status in Puerto Rico (PR).

**Methods:**

We performed a secondary data analysis of women aged 21+ years (n = 469) evaluated at the Anal Neoplasia Clinic (ANC) of the University of Puerto Rico Comprehensive Cancer Center between 2014 and 2023. The outcomes were history of HPV vaccination, anal Pap screening, and cervical Pap screening in the past 3 years. Descriptive statistics were employed to characterize the study population and comparisons were made by HIV status.

**Results:**

Women’s mean age in our study was 50.2 ± 12.1 years; 75.7 % were living with HIV. Among women who were age-eligible for HPV vaccination, 21.7 % reported receiving the HPV vaccine; WLWH were less likely vaccinated than women without HIV (14.7 % vs. 38.7 %, p < 0.01). Prevalence of cervical and anal Pap screening were significantly higher among WLWH (98.6 % and 89.9 %, respectively) than in women without HIV (94.4 % and 47.7 %, respectively). Several risk factors for HPV-related disease were more common among WLWH, such as age at first sexual encounter (≤ 16 years) (54.7 %), more than six lifetime sexual partners (43.1 %), and being a current smoker (23.6 %).

**Conclusions:**

Although we observed high uptake of cervical and anal Pap screening in this clinic-based sample, HPV vaccination was deficient. Future studies should involve developing tailored cancer prevention strategies for WLWH to increase HPV vaccination uptake.

## Highlights

• Women living with HIV are at increased risk of persistent HPV infection and HPV-related cancers.
• A low proportion of age-eligible women (≤45 years old) living with HIV are vaccinated against HPV.
• More than 90 % of women living with HIV underwent cervical and/or anal Pap screening uptake.
• High-risk HPV anal infection and high-grade squamous intraepithelial lesions are commonly found in women living with HIV.
• Tailored interventions are needed to enhance access to HPV-related cancer prevention strategies among women living with HIV.

## Graphical abstract

**Figure:**
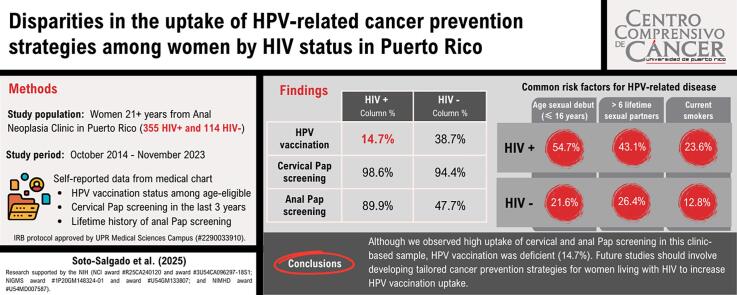

## 1 Introduction

Women living with HIV (WLWH) have a higher risk of persistent infection with high-risk human papillomavirus (HR-HPV) than women without HIV (Castle et al., 2021). Persistent HR-HPV infection can increase the risk of pre-cancerous and cancerous lesions in the cervix and anus (Chiao et al., 2020). Hence, WLWH are at higher risk of developing cervical and anal cancer when compared with women without HIV (Chiao et al., 2020). When race/ethnicity is considered, Hispanic WLHW have a 2-fold higher incidence rate of cervical cancer than non-Hispanic White women, but a similar rate to non-Hispanic Black women (Ortiz et al., 2018). Additionally, women with HPV-related gynecological cancers have an increased risk of secondary anal cancer than women without cancer (Acevedo-Fontánez et al., 2018).

Puerto Rico (PR) has a high HIV burden. According to the 2021 HIV Surveillance Report from the Centers for Disease Control and Prevention, HIV incidence was 10.8 per 100,000 in the United States and 12.6 per 100,000 in PR (Centers for Disease Control and Prevention, 2023). Furthermore, PR is the seventh highest jurisdiction of the United States regarding HIV prevalence (484.2 per 100,000) (Centers for Disease Control and Prevention, 2023). Although anal and cervical cancer are preventable, limited information exists about the uptake of primary prevention strategies (such as the HPV vaccine) and secondary prevention strategies (such as cervical and anal cancer screening) among WLWH in PR. Only one previous study from Soto-Salgado et al. (2024) found a high uptake of cervical cancer screening (91.6 %, Pap test within the past three years) among WLWH in PR using data from a population-based study. Thus, we aimed to estimate 1) the uptake of primary and secondary prevention strategies for cervical and anal cancer, and 2) the presence of risk factors for HPV-related disease among WLWH as compared to women without HIV in a clinic-based sample in PR.

## 2 Methods

We performed a secondary data analysis of the database from the Anal Neoplasia Clinic (ANC) of the University of Puerto Rico Comprehensive Cancer Center. The ANC provides services to patients from all over PR and specializes in the diagnosis and treatment of anal cancer and its precursors, high-grade squamous intraepithelial lesions (HSIL). Women who receive services at the ANC are referred from community HIV clinics or gynecology clinics if they have had abnormal anal cytology results, are at risk of anal cancer, have symptoms that suggest anal cancer, have health concerns about anal cancer, or have a history of HPV-related gynecological disease. Between October 2014 and November 2023, a total of 475 women received services at the ANC, 98.7 % (n = 469) of whom had records that included information on HIV status (355 HIV + and 114 HIV-) and were included in the current analysis. The study was approved by the Institutional Review Board (IRB) of the University of Puerto Rico Medical Sciences Campus (IRB protocol #2290033910).

Data from each patient’s initial visit to the clinic was collected from their medical chart. Information collected included 1) self-reported HPV vaccination status, 2) cervical Pap screening in the last three years, and 3) lifetime history of anal Pap screening. In addition, we describe demographics such as age, marital status, and, for WLWH, years living with HIV. In terms of clinical history, we collected data on history of sexually transmitted diseases (yes or no), as well as anal HR-HPV infection (yes or no to HPV-16, HPV-18, or other HR-HPV), and biopsy-confirmed anal HSIL (normal/low-squamous intraepithelial lesions [LSIL] or HSIL) at the baseline visit. Regarding self-reported behavioral risk factors, we included information on age of first sexual intercourse (≤ 16 years or > 16 years), lifetime number of sexual partners (0–1, 2–5, or 6 or more partners), lifetime history of anal sex (yes or no), smoking history (never, former smoker, or current smoker), alcohol use (ever or never), and illicit drug use (yes or no).

Descriptive statistics were employed to characterize the study population. A Chi-squared test, Fisher’s Exact test, or Student’s *t*-test were performed, as appropriate, to determine differences between selected characteristics and HIV status. Statistical analyses on HPV vaccination were only performed among those who were age-eligible (i.e., women between 21 and 26 years old before 2018, as well as those who turned 45 at any time between 2018 and 2023).

## 3 Results

Table 1 shows the distribution of demographics, clinical characteristics, and self-reported behavioral risk factors for HPV-related disease among women according to HIV status. WLHW were younger than those without HIV (49.3 ± 11.0 and 53.2 ± 14.7, p < 0.01). Also, a higher proportion of WLWH were current smokers (23.6 % vs. 12.8 %, p < 0.01) and used illicit drugs (37.2 % vs. 12.0 %, p < 0.001) as compared to women without HIV. About 54.7 % of the WLHW had their first sexual encounter at 16 years of age or younger and 43.1 % reported having six or more sexual partners in their lifetime, respectively; these proportions were significantly higher than in women without HIV (p < 0.001; Table 1). Approximately 51.0 % of WLWH had engaged in anal intercourse at some point and 58.3 % had a history of sexually transmitted diseases other than HIV; neither of these factors were significantly different from women without HIV (p > 0.05; Table 1). Approximately 37.5 % and 30.9 % of women with and without HIV, respectively, had histologically confirmed anal HSIL (p = 0.25). Also, about 74.0 % and 57.1 % of the women with and without HIV, respectively, had anal high-risk HPV infection (p < 0.01).

**Table 1:** Demographics, behavioral risk factors, and clinical characteristics among women attending the Anal Neoplasia Clinic of the UPRCCC from 2014 to 2023 according to HIV status.

**Characteristics**	**HIV +** **n = 355** **n (col %)**	**HIV –** **n = 114** **n (col %)**	**p-value**
**Demographics**			
Age in years (n = 467)			<0.01^a^
Mean ± SD	49.3 ± 11.0	53.2 ± 14.7	
Median (P25, P75)	50 (41, 57)	54 (42, 66)	
Marital Status (n = 444)			0.79
Partnered	167 (50.3)	58 (51.8)	
Single	165 (49.7)	54 (48.2)	
**Behavioral Risk Factors**			
Smoking (n = 406)			<0.01^a^
Never	122 (41.1)	66 (60.6)	
Previous smoker	105 (35.4)	29 (26.6)	
Current smoker	70 (23.6)	14 (12.8)	
Use of illicit drugs			<0.001^a^
Never	191 (62.8)	95 (88.0)	
Ever	113 (37.2)	13 (12.0)	
Alcohol Use (n = 418)			0.95
Never	86 (27.8)	30 (27.5)	
Ever	223 (72.2)	79 (72.5)	
Age at First Sexual Intercourse (n = 422)			<0.001^a^
≤ 16 years	170 (54.7)	24 (21.6)	
> 16 years	141 (45.3)	87 (78.4)	
Lifetime Number of Sexual Partners (n = 416)			<0.001^a^
0–1	10 (3.3)	17 (15.5)	
2–5	164 (53.6)	64 (58.2)	
6 or more	132 (43.1)	29 (26.4)	
Lifetime Anal Intercourse (n = 469)			0.86
No	174 (49.0)	57 (50.0)	
Yes	181 (51.0)	57 (50.0)	
**Clinical History**			
Body Mass Index (n = 401)			0.34
Underweight/Normal	70 (24.1)	32 (28.8)	
Overweight/Obese	220 (75.9)	79 (71.2)	
Years living with HIV (n = 329)			−
Mean ± SD	17.2 ± 9.6	−	
Median (P25, P75)	17 (9, 25)	−	
History of STDs (n = 435)			0.09
No	135 (41.7)	36 (32.4)	
Yes	189 (58.3)	75 (67.6)	
Anal Histology (n = 388)			0.25
Normal/ LSIL	182 (62.5)	67 (69.1)	
HSIL	109 (37.5)	30 (30.9)	
Anal HR-HPV Infection (n = 276)			<0.01^a^
No	50 (26.0)	36 (42.9)	
Yes	142 (74.0)	48 (57.1)	

Note: Chi-squared test and Student’s *t*-test, as appropriate, were used to calculate p-values unless otherwise specified.

a A statistically significant association was found (p < 0.05).

Table 2 shows the distribution of HPV-related cancer prevention strategies in our sample. Among women who were age-eligible for HPV vaccination, a lower proportion of WLWH had been vaccinated against HPV as compared to women without HIV (14.7 % vs. 38.7 %, p < 0.01). Nearly 98.6 % and 94.4 % of the women with and without HIV, respectively, had a cervical Pap screening within the last 3 years (p = 0.03). Meanwhile, 89.9 % of the WLWH had ever had an anal Pap screening previous to the initial visit to the ANC, as compared to 47.7 % of those without HIV (p < 0.001).

**Table 2:** Primary and secondary cervical and anal cancer prevention strategies uptake at baseline visit among women attending the Anal Neoplasia Clinic at the UPRCCC from 2014 to 2023 according to HIV status.

**Characteristics**	**HIV +** **n = 355** **n (col %)**	**HIV –** **n = 114** **n (col %)**	**p-value**
HPV Vaccine^a^ (n = 106)			<0.01^b^
No	64 (85.3)	19 (61.3)	
Yes	11 (14.7)	12 (38.7)	
History of Cervical Pap Screening (n = 399)			0.03^b^, ^c^
< 3 years	288 (98.6)	101 (94.4)	
≥ 3 years	4 (1.4)	6 (5.6)	
History of Anal Pap Screening (n = 453)			<0.001^b^
Never	35 (10.1)	56 (52.3)	
Ever	311 (89.9)	51 (47.7)	

Note: Chi-squared test was used to calculate p-values unless otherwise specified.

a The analysis included women who met the age eligibility criteria for HPV vaccination: those between 21 and 26 years old before 2018, as well as those who turned 45 at any time between 2018 and 2023.

b A statistically significant association was found (p < 0.05).

c Fisher’s Exact test was used to calculate p-values.

## 4 Discussion

Data about primary and secondary HPV-related cancer prevention strategies among women by HIV status in PR is limited. This information is particularly relevant given the higher burden of HIV/AIDS in PR, where 27 % of the people living with HIV (PLWH) are women (Puerto Rico Department of Health, 2024). Our study reveals that the HPV vaccine uptake was significantly lower among WLWH than in women without HIV (14.7 % and 38.7 %, respectively). Additionally, we observed higher proportions of WLWH undergoing cervical Pap and anal Pap screening (98.6 % and 89.9 %, respectively) as compared to women without HIV.

In PR, HPV vaccination uptake among WLWH remains low although public and most private health insurance plans cover the HPV vaccine for individuals older than 26 years. This may be explained by low knowledge about the efficacy of the HPV vaccine or of the eligibility for vaccination in this age group and lack of provider recommendation, a fact that may affect the decision to vaccinate. Moreover, it is of interest that HPV vaccination was lower among age-eligible WLWH, which are at much higher risk of HPV-related disease, than women without HIV. For instance, in our study, first sexual encounter at a young age, high number of sexual partners, and being a current smoker, known risk factors for HPV infection (Mayo Clinic Staff, 2021), were more prevalent among WLWH, which highlights the importance of the HPV vaccination uptake among WLWH in PR. A higher prevalence of risk factors for HPV-related disease observed in our study was consistent with findings from other researchers (Colón-López et al., 2018, Gursahaney et al., 2019).

Despite the low proportion of HPV vaccination in WLWH than in women without HIV in our sample, both proportions were higher than the 7.3 % of women between 21 and 45 years old from the general population in PR who are vaccinated against HPV, according to the Behavioral Risk Factor Surveillance System in 2014 (unpublished data), the most recent available data for adults of those ages in PR. Nonetheless, it is important to highlight that it was not until late 2018 that the Food Drug Administration approved HPV vaccination up to the age of 45 years (Colón-López et al., 2021). It is also worth emphasizing that our study's data collection period was 2014–2023 and increases in HPV vaccine uptake most likely have occurred with time, similar to what has been reported for HPV awareness among adults aged 21–49 in PR (Castañeda-Avila et al., 2022). However, when compared to other HIV populations, our findings indicate a higher vaccination prevalence than the 4.23 % reported by Boivin & Desai (2024) among PLWH receiving services at the HIV clinic of Hackensack University in the United States. Differences in the proportion of HPV vaccination in our sample groups and other populations could be due to the clinic-based sample evaluated in our study.

The uptake of cervical Pap screening in the ANC among WLWH was higher than that previously reported by Soto-Salgado et al. (2024) for cervical Pap in the past 3 years in PR (91.6 %). The high uptake of cervical Pap screening in our study may reflects proper access to this cancer prevention strategy among women in the study population, irrespective of HIV status. This is important as disparities in access to cancer care have been reported for PLWH in other populations (McGee-Avila et al., 2024). Also, the prevalence of a history of anal Pap screening was considerably higher among WLWH than among women without HIV. The anal Pap screening is not routinely recommended for women without HIV unless they are at high risk for anal cancer (i.e., those with a history of vulvar HSIL or cancer and solid organ transplant) (Stier et al., 2024). The high proportion of women with previous anal Pap screening is also explained by the fact that they come referred to the clinic due to anal concerns.

The prevalence of cervical and anal Pap screening was higher than that reported by Wijayabahu et al. (2019) among PLWH aged 18 + from the Florida Cohort between 2014 and 2017. In that study, 75.3 % of age-eligible WLWH had had a cervical Pap test in the past 3 years and 46.7 % had had an anal Pap test in the past 3 years (Wijayabahu et al., 2019). The difference in the uptake of secondary prevention strategies for cervical and anal cancer in our study compared to other studies may be impacted by our sample, who were referred to a specialized anal neoplasia clinic for their potentially higher risk of anal cancer and thus could be more engaged in healthcare.

### 4.1 Limitations

Our study has some limitations to be considered, including incomplete data on certain characteristics (i.e., anal HPV infection and HPV vaccination) and known behavioral risk factors for HPV infection, as well as a clinic-based sample design that could make our study participants show higher rates of cancer screening than those not receiving HIV care. Although women have been receiving services since the ANC began operating in 2014, data on HPV vaccination began to be collected in mid-2015. This data limitation regarding HPV vaccination may have excluded a potentially relevant group, not allowing us to generalize our results to a larger population. Also, measurement errors might have resulted from recall or social desirability biases for self-reported data.

## 5 Conclusion

Despite limitations, our study is the first to compare primary and secondary HPV-related cancer prevention strategies, as well as risk factors for HPV-related disease among a clinic-based high-risk sample of women in PR. It evidences a high uptake of cervical Pap screening in women within the clinical setting in PR, irrespective of HIV status, suggesting no disparity in screening access and provides a recent view of HPV vaccination in both study groups aged 21–45 years, from a clinic-based perspective. Our findings contribute to our understanding of HPV-vaccine underutilization and the increased prevalence of risk factors for HPV-related disease among WLWH on the island, even though these women may be more regularly engaged in healthcare than the general population.

Enhancing the uptake of HPV-related cancer screening and HPV vaccination is a key step for cancer prevention efforts among WLWH in PR. Given the high burden of HPV-related risk factors in this population, future studies should involve a population-based representative sample. This approach will aid in identifying barriers and facilitators to HPV vaccination and HPV-related cancer screening. This information will be used to develop effective and tailored interventions to improve access to HPV-related cancer prevention strategies for WLWH, taking into account the newly established guidelines for anal cancer screening, the updated guidelines for cervical cancer screening, and current recommendations on HPV vaccination (Panel on Guidelines for the Prevention et al., 2024). Moreover, educating WLWH and healthcare providers, enhancing community outreach, and providing economic resources to cover the costs of the HPV vaccine may improve the uptake of this cancer prevention strategy.

## CRediT authorship contribution statement

**Marievelisse Soto-Salgado:** Writing – review & editing, Writing – original draft, Supervision, Methodology, Investigation, Formal analysis, Conceptualization. **Melisa Ramos-Sepúlveda:** Writing – review & editing, Writing – original draft, Visualization, Formal analysis. **Jeslie M. Ramos-Cartagena:** Writing – review & editing, Data curation. **Maribel Tirado-Gómez:** Writing – review & editing, Investigation. **Humberto M. Guiot:** Writing – review & editing, Investigation. **Cristina Muñoz:** Writing – review & editing, Validation, Investigation. **Lorena González-Sepúlveda:** Writing – review & editing, Validation. **Ana Patricia Ortiz:** Writing – review & editing, Investigation, Conceptualization.

## Declaration of Competing Interest

Dr. Ortiz has been a consultant for Merck Inc. and has received grants from the company related to work outside of this submission. No additional competing interests or personal relationships that could have appeared to influence the work reported in this paper were reported by the rest of the authors.
